# IgG and IgA autoantibodies against L1 ORF1p expressed in granulocytes correlate with granulocyte consumption and disease activity in pediatric systemic lupus erythematosus

**DOI:** 10.1186/s13075-021-02538-3

**Published:** 2021-05-29

**Authors:** Kennedy C. Ukadike, Kathryn Ni, Xiaoxing Wang, Martin S. Taylor, John LaCava, Lauren M. Pachman, Mary Eckert, Anne Stevens, Christian Lood, Tomas Mustelin

**Affiliations:** 1 Division of Rheumatology, University of Washington, 750 Republican Street, Room E507, Seattle, WA 98109 USA; 2 Massachusetts General Hospital, Boston, and Whitehead Institute, Cambridge, MA USA; 3 The Rockefeller University, New York, NY USA; 4 European Research Institute for the Biology of Ageing, University Medical Center Groningen, Groningen, The Netherlands; 5 Ann & Robert H. Lurie Children’s Hospital of Chicago, and Northwestern University Feinberg School of Medicine, Chicago, IL USA; 6 Seattle Children’s Hospital, Seattle, WA 98105 USA; 7 Seattle Children’s Research Institute, Seattle, WA 98101 USA; 8 Current affiliation: Jansen Research and Development LLC, Malvern, PA USA

**Keywords:** Pediatric lupus erythematosus, Long interspersed nuclear element, Retrotransposon, Autoantibodies, Neutrophils

## Abstract

**Background:**

Most patients with systemic lupus erythematosus (SLE) have IgG autoantibodies against the RNA-binding p40 (ORF1p) protein encoded by the L1 retroelement. This study tested if these autoantibodies are also present in children with pediatric SLE (pSLE) and if the p40 protein itself could be detected in immune cells.

**Methods:**

Autoantibodies in the plasma of pSLE patients (*n* = 30), healthy children (*n* = 37), and disease controls juvenile idiopathic arthritis (JIA) (*n* = 32) and juvenile dermatomyositis (JDM) (*n* = 60), were measured by ELISA. Expression of p40 in immune cells was assessed by flow cytometry. Markers of neutrophil activation and death were quantitated by ELISA.

**Results:**

IgG and IgA autoantibodies reactive with p40 were detected in the pSLE patients, but were low in healthy controls and in JIA or JDM. pSLE patients with active disease (13 of them newly diagnosed) had higher titers than the same patients after effective therapy (*p* = 0.0003). IgG titers correlated with SLEDAI (*r* = 0.65, *p* = 0.0001), ESR (*r* = 0.43, *p* = 0.02), and anti-dsDNA antibodies (*r* = 0.49, *p* < 0.03), and inversely with complement C3 (*r* = -0.55, *p* = 0.002) and C4 (*r* = -0.51, *p* = 0.006). p40 protein was detected in a subpopulation of CD66b^+^ granulocytes in pSLE, as well as in adult SLE patients. Myeloperoxidase and neutrophil elastase complexed with DNA and the neutrophil-derived S100A8/A9 were elevated in plasma from pSLE patients with active disease and correlated with anti-p40 autoantibodies and disease activity.

**Conclusions:**

Children with active SLE have elevated IgG and IgA autoantibodies against L1 p40, and this protein can be detected in circulating granulocytes in both pediatric and adult SLE patients. P40 expression and autoantibody levels correlate with disease activity. Markers of neutrophil activation and death also correlate with these autoantibodies and with disease activity, suggesting that neutrophils express L1 and are a source of p40.

**Supplementary Information:**

The online version contains supplementary material available at 10.1186/s13075-021-02538-3.

## Background

Type I interferons (IFNs) play a central role in antiviral immunity [1]. They are also increased in a number of diseases, such as systemic lupus erythematosus (SLE) [2–5], dermatomyositis [6], and Sjögren’s syndrome [7], which are characterized by autoimmunity against nucleic acids and proteins that associate with them. The cellular sources of these pathogenic nucleic acids and how they provoke autoimmunity are still incompletely understood [8].

A number of DNA and RNA sensors have been discovered in recent years [9, 10]. They are widely expressed (i.e., also outside of the immune system) and they play key roles in the cell-intrinsic defense against viral infection, where they detect non-self (e.g., viral) DNA or RNA and relay this information to the innate and adaptive immune systems through the production of predominantly IFNβ (and less IFNα) and upregulating MHC and other immune recognition molecules. It is now becoming evident that these sensors can be aberrantly activated also in autoimmune or genetic disorders that are characterized by high type I IFNs [11, 12]. Elevated cyclic guanosine adenosine phosphate [13], indicative of activated DNA sensor cGAS, and oligomerized MAVS [14], a consequence of activation of the RNA sensors MDA5 or RIG-I, are detectable in a subset of SLE patients. The nature of the DNA and RNA species that are responsible for triggering these sensors remains unclear [8].

One possible source of cytosolic DNA is the reverse transcriptase encoded by the second open-reading frame (ORF2p) of the long interspersed nuclear element (LINE-1; L1), sequences of which constitute 17% of our genome [15–18] and which are detectably expressed in patients with SLE or Sjögren’s syndrome [19, 20]. ORF2p is a reverse transcriptase [21, 22] that, in addition to its own mRNA, can use many cellular RNAs including Alu transcripts as templates [17, 18] to generate RNA:DNA hybrids and double-stranded DNA that trigger IFNβ production through cGAS activation [23].

We recently reported [24] that adult SLE patients have IgG autoantibodies against the protein encoded by the first open-reading frame of L1, the RNA-binding 40-kDa ORF1p (p40). The anti-p40 autoantibodies correlated with disease activity and serological measures of the disease. Here, we extend these findings to pediatric SLE patients, asking whether retroelement activation is an early event in development of autoimmunity. With considerably lower baseline anti-p40 IgG in healthy children than seen in healthy adults, we find that the correlations between anti-p40 IgG autoantibodies and disease measures are more striking than in adult SLE patients. Pediatric lupus patients, but not adult SLE patients, also had IgA autoantibodies in circulation against p40. Furthermore, a subset of p40-expressing granulocytes were present in pediatric as well as adult SLE patients. The percentage of granulocytes expressing p40 correlated with disease activity. We also find that pSLE patients have elevated markers of neutrophil death, which also correlated closely with anti-p40 antibodies. Taken together, our data support the novel finding that L1 retroelement expression in neutrophils is an early event in SLE.

## Patients and methods

### pSLE patients, JIA patients, and healthy controls

A cohort of pSLE patients (*n* = 30) were recruited at Seattle Children’s Hospital, from whom two plasma samples were collected, one during a period of active disease and another when the disease was inactive (SLEDAI ≤ 4), with a range of 1–67 (average 15.5) months between the two samples. In 13 cases, the first sample was taken at the time of diagnosis. The clinical characteristics of this cohort are summarized in Table 1. Exclusion criteria included severe anemia and inability to consent. A cohort of JIA patients (*n* = 32) and healthy age-matched individuals (*n* = 17) were also included (Table 1). An additional exclusion criterion for healthy subjects was any history of immune-mediated disorder. The study was approved by the Seattle Children’s Research Hospital Human Subjects Committee. Informed written consent was obtained from the parents or guardians of all participants according to the Declaration of Helsinki.

**Table 1:** pSLE patients, JIA patients, and healthy controls

**Characteristic**	**Active pSLE (*n*** **= 30) ± SD**	**Inactive pSLE (*n*** **= 30) ± SD**
Age	14.7 ± 2.9	14.9 ± 2.6
Laboratory measures:		
C3	77.3 ± 34.1	99.9 ± 20.0
C4	9.1 ± 7.8	16.2 ± 8.1
ESR	39.1 ± 33.6	16.1 ± 14.2
ANA	29	29
Anti-dsDNA pos.	20	15
Disease activity and ACR criteria:		
SLEDAI	10.6 ± 4.8	2.4 ± 1.8
SLEDAI = 0	1	9
Arthritis	23	
Oral ulcers	13	
Rash		
Nephritis	12	
Pleuritis/pericarditis	9	
CNS symptoms	1	
Photosensitivity	7	
Current treatment:		
None	10	3
Hydroxychloroquine	16	24
Steroid	16	22
Other DMARD	13	19
Biologic only	1	1
**Characteristic**	**JIA (*n*** **= 32) ± SD**	**Healthy children (*n*** **= 37) ± SD**
Age	13.9 ± 2.2	12.3 ± 4.4
JIA subtype:		
Persistent oligoarticular	7	
Extended oligoarticular	6	
Polyarticular RF−	17	
Polyarticular RF+	2	
Active disease	24	na
Active joints	3.3 ± 3.6	na
Laboratory measures:		
RF positive	2	nd
ACPA positive	2	nd
ESR	11.2 ± 9.5	nd
CRP	0.9 ± 0.9	nd
Current treatment:		
None or NSAID only	7	na
Methotrexate only	8	na
Other DMARD only	3	na
Biologic only	6	na
DMARD + biologic	8	na

### Juvenile dermatomyositis (JDM) patients and healthy controls

A cohort of JDM patients (*n* = 60) and healthy controls (*n* = 20) were recruited at Ann & Robert H. Lurie Children’s Hospital of Chicago, Northwestern University Feinberg School of Medicine, Chicago, IL, Institutional Review Board of which approved the study. Informed written consent was obtained from the parents or guardians of all participants according to the Declaration of Helsinki. The healthy controls from this hospital were combined with the ones from Seattle Children’s Hospital giving a total of *n* = 37 control samples for the ELISAs.

### Adult SLE patients

A cohort of adult patients with SLE (*n* = 10) were recruited through the University of Washington, Division of Rheumatology Biorepository. The study was approved by the University of Washington Institutional Review Board. Informed written consent was obtained from all participants according to the Declaration of Helsinki.

### ELISAs

As described before [24], 3.3 μg/ml of purified p40 protein was adsorbed onto 96-well polystyrene plates in 0.1 M bicarbonate (pH 9.6) buffer overnight, washed in phosphate-buffered saline with 0.05% Tween, and blocked in 2% bovine serum albumin (BSA) in phosphate-buffered saline for 2 h. Plasma was added at 0.5% in blocking buffer (with BSA) for overnight incubation at 4 °C, washed extensively, and then incubated with 1:2000 dilution of horseradish peroxidase-conjugated anti-human IgG. The reaction was then washed and developed with TMB, with the color reaction terminated with 2 N sulfuric acid, and the absorbance measured at 450 nm using a plate reader. Autoantibodies against p40 of IgM, IgA, and IgE class were measured using the same protocol, but with secondary antibodies specific for human IgM, IgA, or IgE.

### Neutrophil activation and death assays

Levels of calprotectin (S100A8/A9) were analyzed using a commercial ELISA kit according to the manufacturer’s instruction (R&D Systems). Levels of MPO-DNA and NE-DNA complexes were analyzed using sandwich ELISAs as described before [25, 26]. Briefly, a 96-well microtiter plate was coated with capture antibody, 4 μg/mL, followed by blocking with 1% BSA. After blocking, plasma samples, diluted 1/10, were added and incubated overnight. For detection, anti-dsDNA-HRP antibody (diluted 1/100, Roche Diagnostics) was added for 2 h. The reaction was developed with 3,3′,5,5′-tetramethylbenzidine (TMB, BD Biosciences), and ended by the addition of 2 N sulfuric acid. Absorbance was measured at 450 nm by a plate reader (Synergy, BioTek). Pure MPO-DNA and NE-DNA complexes of known concentration were used as standard curve.

### Immune cell isolation

Polymorphonuclear (PMN) and peripheral blood mononuclear cells (PBMC) were isolated from freshly drawn venous blood by gradient centrifugation on PolymorphPrep according to the manufacturer’s instructions. Cells were washed and suspended in Hanks’ buffered salt solution at 10^7^/ml.

### Immunoblotting

PMN or PBMC were lysed by mixing 10^7^ cells in 100 μl with an equal volume of twice concentrated SDS sample buffer, heated at 95 °C, and clarified by centrifugation. In total, 50 μl (2.5 × 10^6^ cell equivalents) samples were resolved by SDS gel electrophoresis and transferred to nitrocellulose membranes, which were blocked in 1% bovine serum albumin in Tris-buffered saline. Filters were incubated with 4H1 anti-p40 mAb diluted 1:1000 in blocking buffer overnight, washed extensively in blocking buffer with 0.2% Tween-20, and developed with horseradish-conjugated anti-mouse antibody and enhanced chemiluminescence detection.

### Flow cytometry

Cells were washed twice in phosphate-buffered saline (PBS) with 1% bovine serum albumin and 1% mouse serum and then stained with a mixture of antibodies against surface antigens: anti-CD66b (PE/Cy7-labeled, Biolegend #305115) at 1:50, anti-CD16 (PerCP-labeled, clone 3G8 Biolegend #302029) at 1:200, anti-CD14 (PE-labeled anti-human CD14 antibody clone 63D3 (Biolegend #367103) at 1:200, and anti-CD19 (APC/Cyanine7-conjugated, clone HIB19, Biolegend #302218) at 1:200, anti-CD15 (PerCP-conjugated, clone W63D, Biolegend #323018) at 1:200 in PBS with 1% BSA and 1% mouse serum for 30 min at 4 °C in the dark. The cells were washed twice, fixed in 1% paraformaldehyde in PBS, permeabilized in 200 μl 0.1% Tween in PBS for 30 min at room temperature, washed and resuspended in 50 μl the same buffer with 1:500 diluted anti-L1 p40 (clone 4H1, EMD Millipore MABC1152) conjugated to APC using Mix-n-Stain™ Fluorescent Protein & Tandem Dye Antibody Labeling Kits (Biotium #92306). After 30 min at 4 °C in the dark, the cells were washed twice with PBS, 1% BSA, and 1% mouse serum and resuspended in 200 μl of this buffer for analysis on a CytoFLEX Flow Cytometer (Beckman Coulter).

### Statistical analyses

For non-paired sample sets with non-Gaussian distribution, Mann-Whitney U test, and Spearman’s correlation test were used, as applicable. For paired sample sets, Wilcoxon matched-pair signed rank test was used. As a cutoff for positivity, the 95th percentile of the healthy controls was used. GraphPad Prism (version 8.4.3) and IBM SPSS were used for the analyses. All analyses were considered statistically significant at *p* < 0.05.

## Results

### IgG autoantibodies against L1 p40 are elevated in pSLE, compared to healthy children, JIA, and JDM

**Fig. 1:**
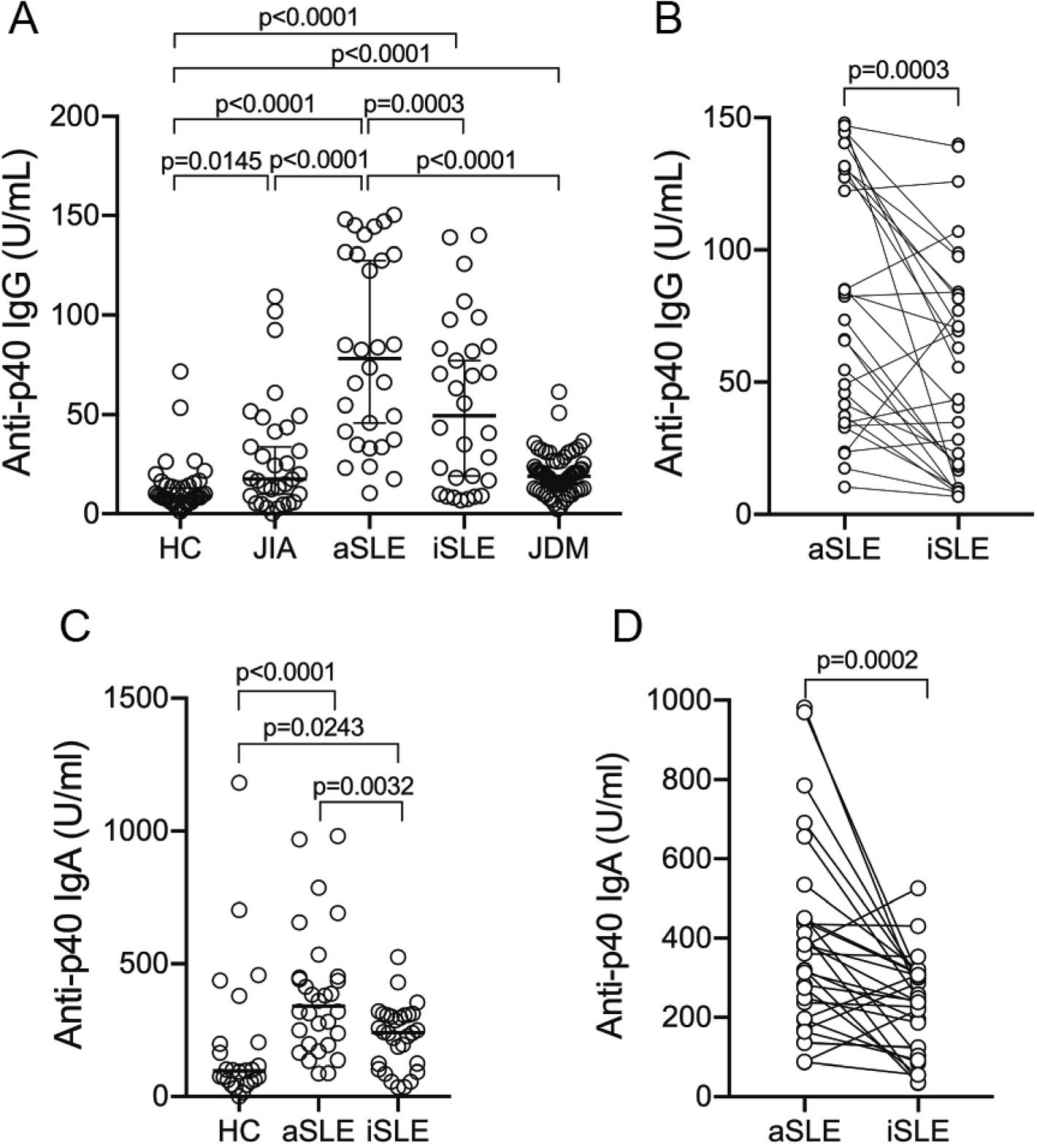
Plasma from pediatric SLE patients contain IgG autoantibodies reactive with L1 p40 protein. **A** Quantitation by ELISA of anti-p40 IgG in the plasma of healthy control children (HC; *n* = 37) or patients with juvenile idiopathic arthritis (JIA; *n* = 32) or pediatric SLE patients before treatment (active, aSLE; *n* = 30) or the same patients after treatment (inactive, iSLE; *n* = 30). **B** Comparison of anti-p40 IgG reactivity in each pSLE patient before and after treatment. **C** Quantitation of anti-p40 IgA in the plasma of HC children and pSLE patients before or after treatment. **D** Comparison of anti-p40 IgA reactivity in each pSLE patient before and after treatment

As most adult SLE patients have IgG autoantibodies that recognize ORF1p/p40 encoded by L1 that can be detected both by immunoblotting and ELISA [24], we used the latter assay to quantitate anti-p40 reactive IgG in pSLE patients. At the time of diagnosis and with active disease, the 30 pSLE patients had much higher anti-p40 reactivity than healthy controls, JIA, and JDM patients (*p* < 0.0001) (Fig. 1A). Using the 95th percentile of the healthy controls as a cutoff for a “normal” range, 26 (87%) of the pSLE patients were above this cutoff, compared to only 2 of the healthy children. JIA patients showed a slight increase with 12 (38%) of patients having anti-p40 titers above this cutoff, but the difference compared to healthy controls was statistically significant at *p* = 0.0145. Children with JDM had only slightly higher titers than those of healthy children.

### Anti-p40 autoantibody levels are higher in patients with active disease

Anti-p40 reactivity was significantly (*p* = 0.0003 by Wilcoxon matched-pair signed rank test) reduced in the second plasma sample from the pSLE patients taken either after several months of therapy, to which many of them had responded well and lowered their SLEDAI score to ≤ 4 (Fig. 1A), or at a time of inactive disease before a subsequent flare. In 22 (73%) of the patients, the titers were reduced, in 4 they were essentially unchanged, and in 4 they were somewhat increased (Fig. 1B). Nevertheless, 19 (63%) of the patients still had anti-p40 titers above the 95th percentile of the healthy controls, a statistically significant difference (*p* < 0.0001) to this control group.

### Anti-p40 autoantibodies of IgA class are also elevated, but not IgM or IgE

To quantitate autoantibodies of other classes than IgG, we used different Ig class-specific secondary antibodies in the ELISA and found that the pSLE patients with active disease had elevated IgA reactivity (*p* < 0.0001), as did the patients with inactive diseases (*p* = 0.0032; Fig. 1C) compared to healthy children. In 25 patients, the titers were lower when their disease was inactive, while it was unchanged in 2 and somewhat increased in 3 (Fig. 1D). These differences were statistically significant (*p* = 0.0002 by Wilcoxon matched-pair signed rank test). In contrast, recognition of p40 by IgM antibodies was similar between the groups and IgE were generally very low (data not shown).

### Anti-p40 autoantibodies correlate with disease activity and complement consumption

**Fig. 2:**
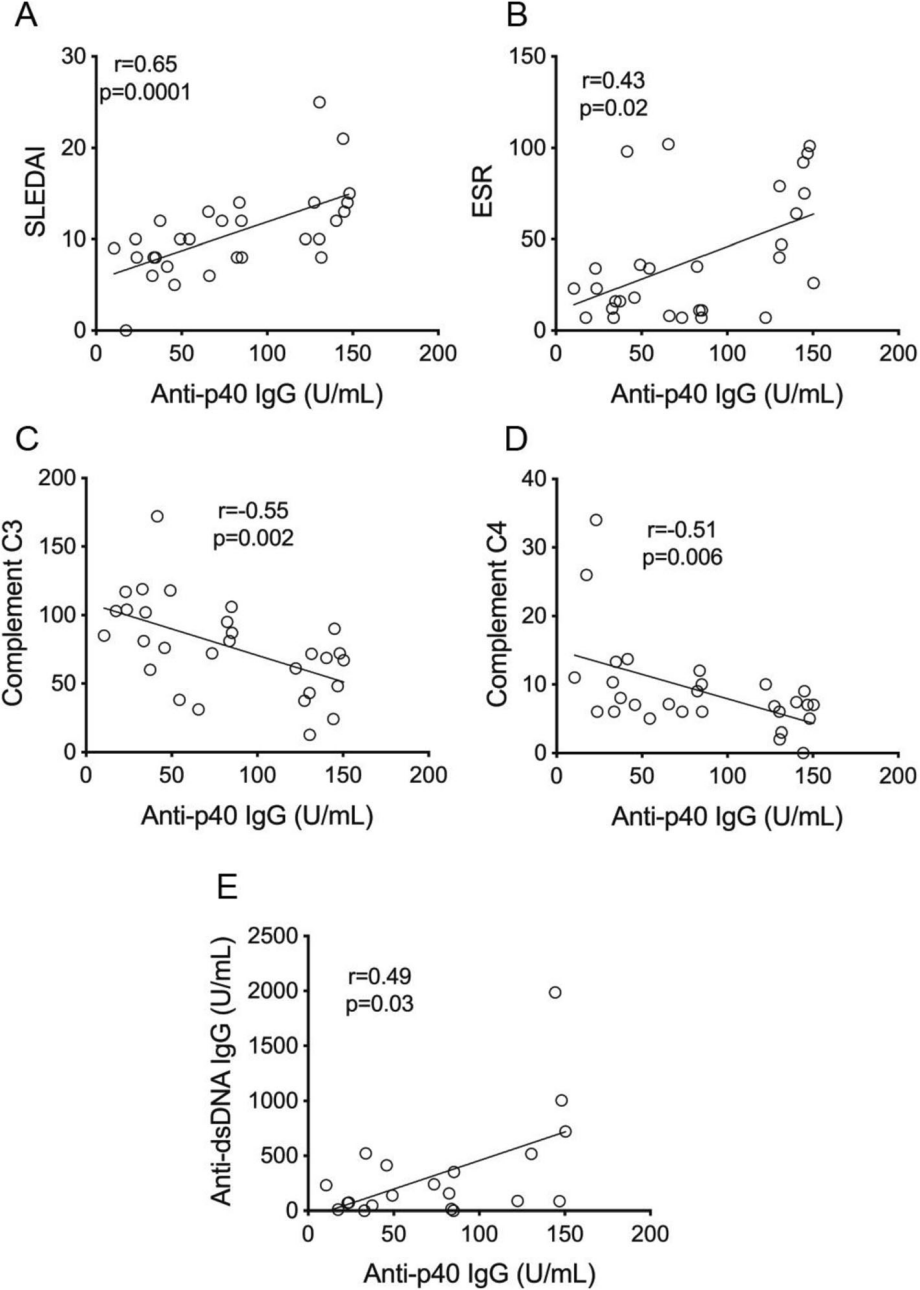
Correlations of autoantibodies against L1 p40 with markers of disease activity. **A** Correlation between anti-p40 autoantibodies and SLEDAI in 29 of the 30 pediatric SLE patients with active disease before treatment. **B** Correlation with erythrocyte sedimentation rate (ESR) in 29 of the 30 pediatric SLE patients. **C** Inverse correlation with complement C3 levels in 29 of the 30 pediatric SLE patients. **D** Inverse correlation with complement C4 levels in 28 of the 30 pediatric SLE patients. **E** Correlation between anti-p40 autoantibodies and anti-dsDNA autoantibody levels in 20 of the 30 pediatric SLE patients with active disease before treatment. Statistical significance by Mann-Whitney U test and correlation by Spearman’s correlation test

The IgG autoantibody titers also correlated with the SLE disease activity index (SLEDAI; *r* = 0.65, *p* = 0.0001) (Fig. 2A), erythrocyte sedimentation rate (ESR; *r* = 0.43, *p* = 0.02) (Fig. 2B), complement C3 and C4 consumption (*r* = − 0.55, *p* = 0.002 and *r* = − 0.51, *p* = 0.006, respectively) (Fig. 2C, D), and anti-dsDNA antibodies (*r* = 0.49, *p* = 0.03) (Fig. 2E). Collectively, these data indicate that higher anti-p40 IgG levels tend to accompany active disease. Anti-p40 IgA levels correlated positively with ESR (*r* = 0.445, *p* = 0.026), but did not correlate in a statistically significant manner with other measures of disease activity.

### Detection of p40 in patient immune cells

**Fig. 3:**
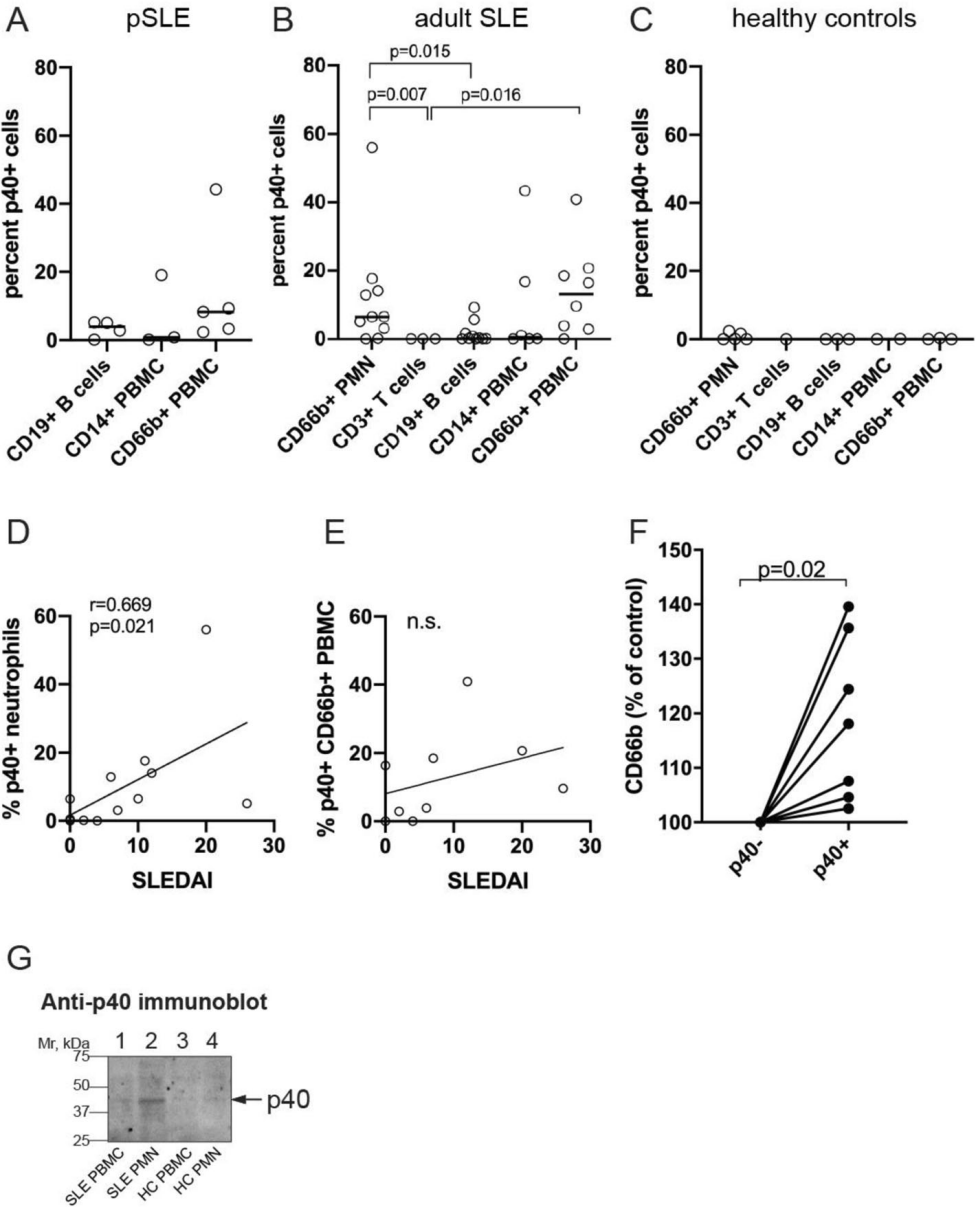
Presence of p40 protein in a subset of immune cells. **A** Summary of flow cytometry data in pSLE patients. **B** Summary of flow cytometry data in adult SLE patients. **C** Summary of flow cytometry data in healthy controls (HC). **D** Scatter plot of percent p40-positive CD66b+ cells in the PMN fraction versus SLEDAI at the time of blood draw in adult SLE patients. **E** Scatter plot of percent p40-positive CD66b+ cells in the PBMC fraction versus SLEDAI at the time of blood draw in adult SLE patients. **F** Relative expression of CD66b in neutrophils expressing p40 (p40+) compared to those without detectable p40 (p40−). **G** Anti-p40 mAb immunoblot of PBMC and PMN lysates from SLE and HC, as indicated. Identical cell equivalents of each (2.5 × 10^6^) were loaded

We first searched for detectable p40 in leukocyte linages in the PBMC from 5 patients with pSLE and found that all of them had detectable p40 in a subset that varied from 2.4 to 44.2% (mean 16.1 ± 17.6%; standard deviation, SD) of their CD66b+ granulocytes, while much smaller fractions of their CD19+ B cells (0–5.2%) or CD14+ monocytes (0–19%) were positive compared to control antibody-stained cells analyzed by flow cytometry (Fig. 3A; gating strategy and representative results are shown in supplemental Fig. S1). To better explore this finding, we recruited 10 adult SLE patients and 5 healthy adult controls and analyzed their PMN and PBMC fractions by flow cytometry using more lineage markers. These experiments revealed that p40 was detectable in 12.2 ± 16.6% (range 0–56 %) of CD66b + cells in the PMN fractions (Fig. 3B). These values are statistically different from those in T cells (*p* = 0.007) and B cells (*p* = 0.015). In the lower-density PBMC fraction, a similar portion (14.1 ± 13.3%, range 0–40.9%) of the CD66b + cells were also positive for p40 (Fig. 3B), a statistically significant difference from the T cells (*p* = 0.016). Only two patients had detectable p40 in their B cells and none in their T cells, while the CD14+ cells in the PBMC fraction, which include monocytes and granulocytes, were positive in two patients (43.4% and 16.7%, respectively). In healthy controls, all leukocyte lineages were negative for p40 (Fig. 3C). These data indicate that neutrophils, particularly considering that they constitute approximately half of all immune cells in circulation, are the predominant cells expressing p40 protein in SLE patients, but that the number of positive cells varied broadly. Patients with the highest SLEDAI scores at the time of blood draw correlated with the highest portion of p40-positive neutrophils in the PMN (*r* = 0.669, *p* = 0.021) (Fig. 3D). A trend towards a similar correlation was seen between SLEDAI and the portion of p40-positive CD66b+ cells in the PBMC fractions (Fig. 3E), but this trends did not meet statistical significance.

The median fluorescence intensity of CD66b staining of neutrophils with detectable p40 was somewhat higher (*p* = 0.02) than of neutrophils without p40 in 7 of the p40-expressing patients (Fig. 3F), suggesting that the presence of p40 may be associated with a more activated neutrophil phenotype. As an independent validation, immunoblotting of cell lysates showed that the PMN fraction from SLE patients had more p40 than the PBMC fraction, while healthy controls were negative (Fig. 3G).

### Markers of neutrophil activation and NETosis are elevated in pSLE patients

**Fig. 4:**
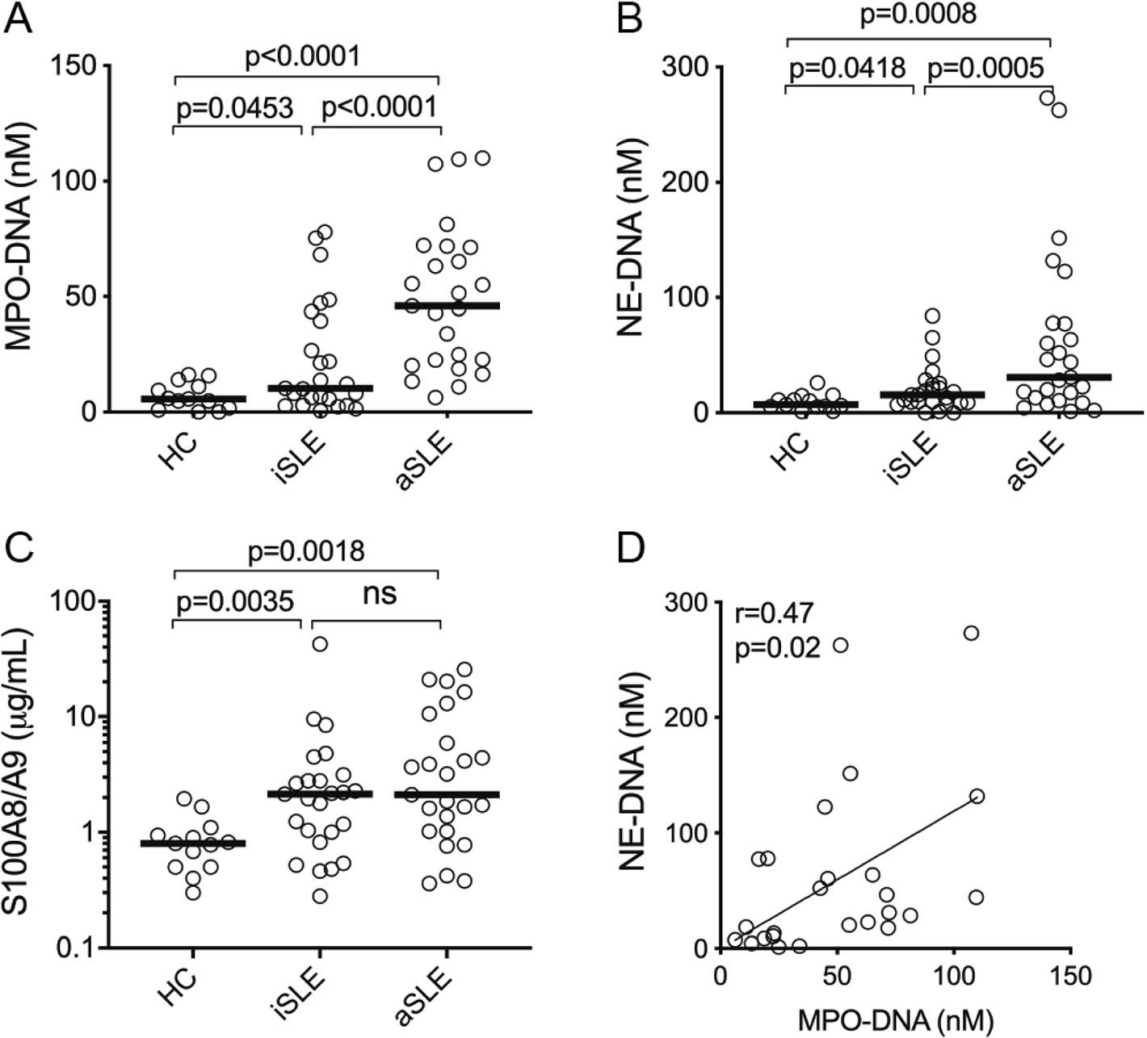
Markers of neutrophil death in pSLE patients and healthy controls. **A** Levels of MPO-DNA complexes in healthy controls (HC) and pediatric lupus patients with inactive (iSLE) and active (aSLE) disease. **B** Levels of NE-DNA complexes in the same patient groups. **C** Levels of calprotectin (S100A8/A9) in the same patient groups. **D** Correlation between MPO-DNA and NE-DNA complexes in patients with active disease. Statistical significance by Mann-Whitney U test and Wilcoxon, and correlation by Spearman’s correlation test

Because p40 appears to be expressed predominantly in neutrophils in both the pediatric and adult SLE patients, and perhaps more so in activated neutrophils, we measured markers for neutrophil activation and NETosis, which have been developed in our lab [26–29]. Myeloperoxidase (MPO) and/or neutrophil elastase (NE) in complex with free DNA in plasma reflect death of neutrophils, including (and perhaps mostly) by NETosis [25]. A second marker, levels of the S100A8/A9 proteins (also known as calprotectin), which reflects neutrophil activation was also measured. As shown in Fig. 4A–C, these markers were all elevated in the plasma of the 30 pSLE patients with active disease, as well as to a lower extent in samples taken from the patients during inactive disease. The levels of MPO-DNA and NE-DNA complexes correlated with each other both during active disease (*r* = 0.47, *p* = 0.018) (Fig. 4D) and during inactive disease (*r* = 0.52, *p* = 0.008) when the values were lower (not shown).

### Anti-p40 autoantibodies correlate with markers of neutrophil death and activation

**Fig. 5:**
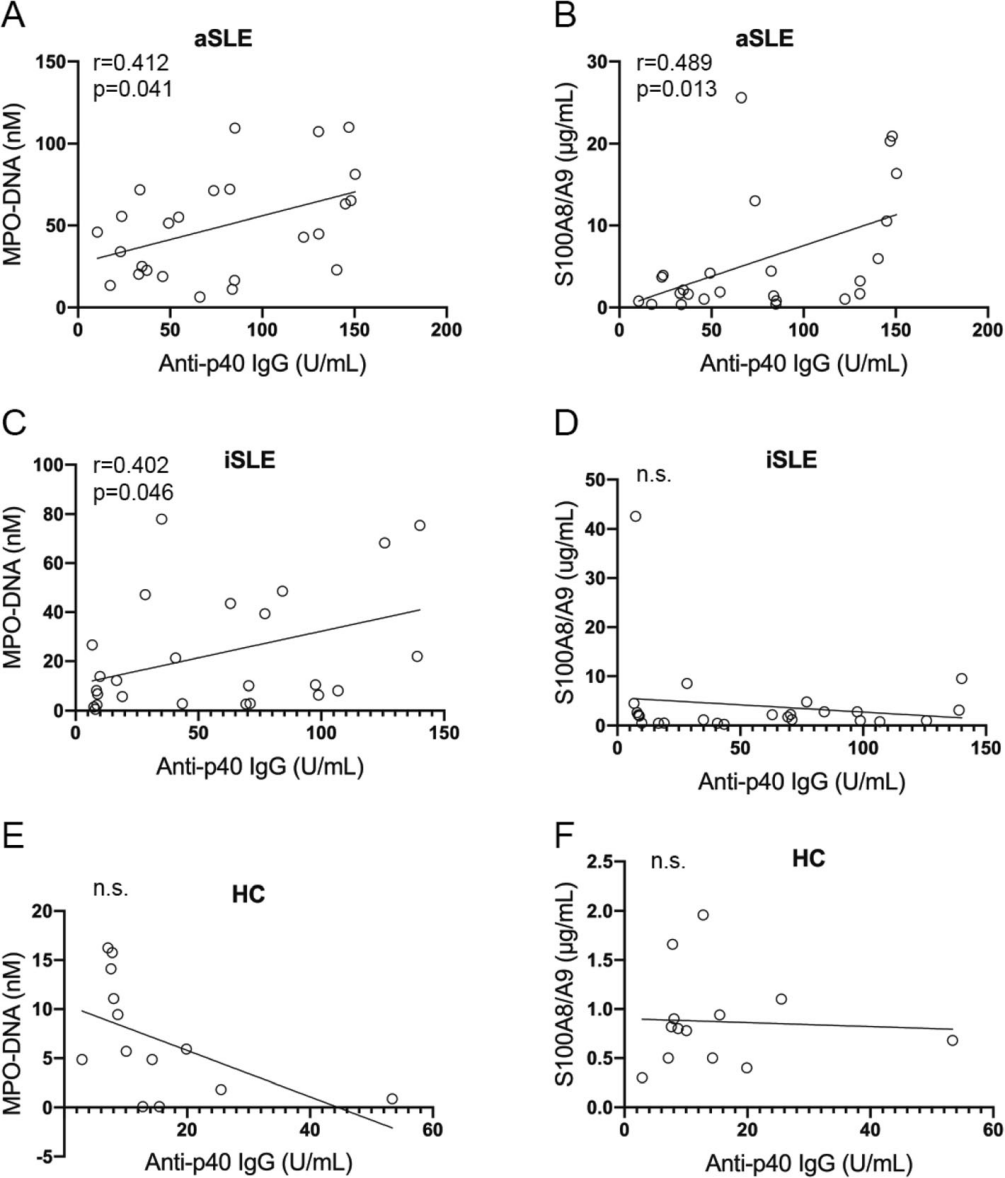
Correlations between IgG anti-p40 autoantibodies and markers of neutrophil death in pSLE patients with active disease and healthy controls. **A** Correlation between levels of MPO-DNA complexes and anti-p40 IgG antibodies in active pSLE patients. **B** Correlation between levels of S100A8/A9 and anti-p40 IgG antibodies in active pSLE patients. **C** Correlation between levels of MPO-DNA complexes and anti-p40 IgG antibodies in inactive pSLE patients. **D** Lack of correlation between levels of calprotectin and anti-p40 IgG antibodies in inactive pSLE patients. **E** Lack of correlation between levels of MPO-DNA complexes and anti-p40 IgG antibodies in healthy controls. **F** Lack of correlation between levels of calprotectin and anti-p40 IgG antibodies in healthy controls.

In pSLE patients with active disease, the titers of anti-p40 autoantibodies of IgG class correlated with MPO-DNA complexes (*r* = 0.412, *p* = 0.041) (Fig. 5A) and S100A8/A9 (*r* = 0.489, *p* = 0.013) (Fig. 5B). However, in inactive disease, only MPO-DNA complexes, but not S100A8/A9, correlated with anti-p40 IgG autoantibodies (Fig. 5C, D).

**Fig. 6:**
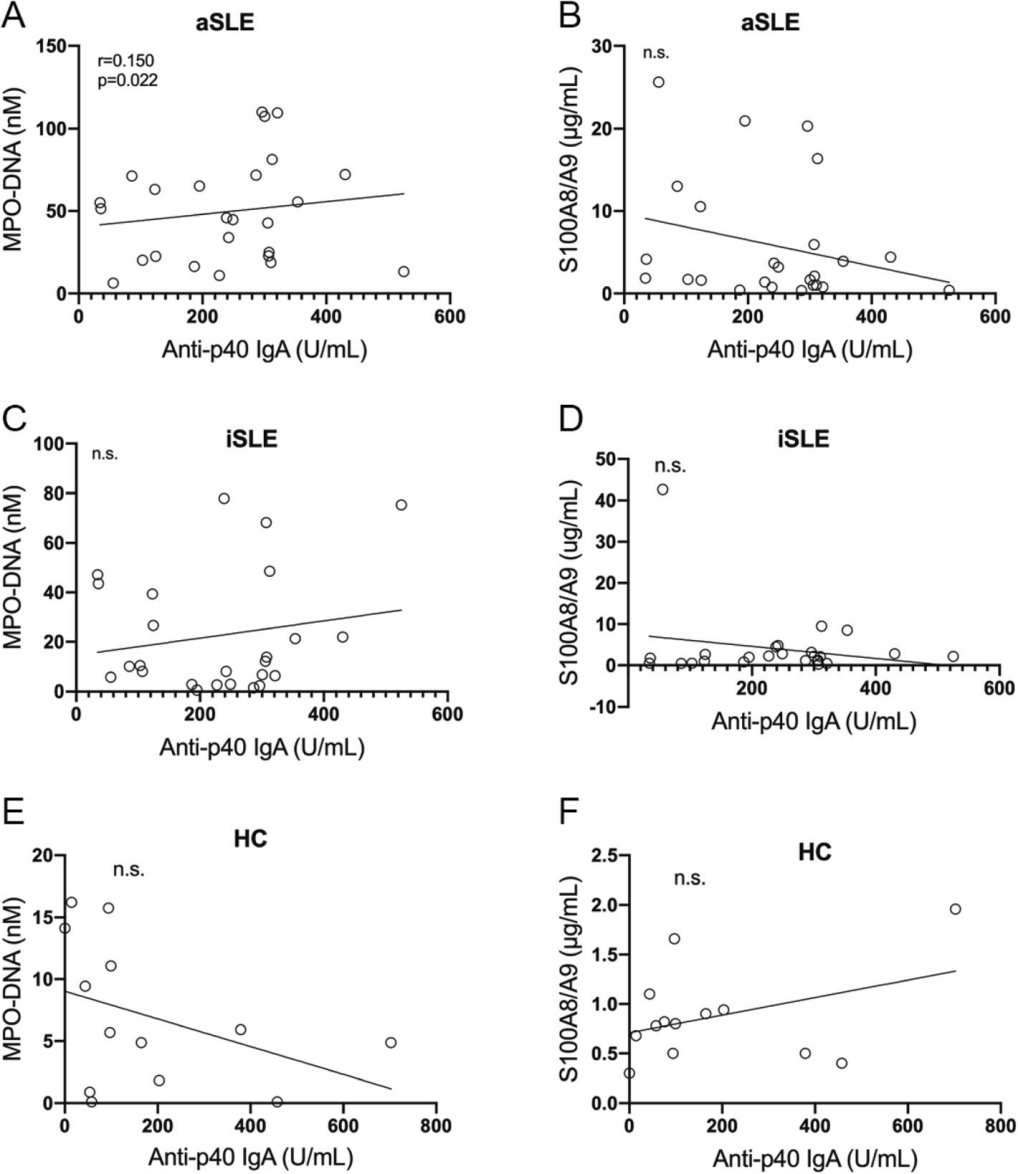
Correlations between IgA anti-p40 autoantibodies and markers of neutrophil death in pSLE patients with active disease and healthy controls. **A** Correlation between levels of MPO-DNA complexes and anti-p40 IgA antibodies in active pSLE patients. **B** Lack of correlation between levels of calprotectin and anti-p40 IgA antibodies in active pSLE patients. **C** Lack of correlation between levels of MPO-DNA complexes and anti-p40 IgA antibodies in inactive pSLE patients. **D** Lack of correlation between levels of calprotectin and anti-p40 IgA antibodies in inactive pSLE patients. **E** Lack of correlation between levels of MPO-DNA complexes and anti-p40 IgA antibodies in healthy controls. **F** Lack of correlation between levels of calprotectin and anti-p40 IgA antibodies in healthy controls

Anti-p40 IgA autoantibodies only correlated statistically significantly with MPO-DNA complexes (*r* = 0.15, *p* = 0.022) in active SLE (Fig. 6A). In healthy controls, all neutrophil markers and autoantibodies were low and did not correlate with each other in a statistically significant manner (Fig. 5E, F; Fig. 6E, F).

## Discussion

We report several novel findings in children with recent-onset lupus, which may be connected with each other. First, that autoantibodies against p40/ORF1p encoded by the L1 retroelement are prevalent in pediatric patients with SLE with active and recently diagnosed disease, compared to those with inactive disease or healthy controls. In contrast, pediatric patients with JIA or JDM had considerably lower reactivity with p40. Similarly, while adult SLE patients also have anti-p40 autoantibodies, patients with scleroderma [24] or rheumatoid arthritis (our unpublished observation) are generally low or negative compared to healthy controls, with only a few individual exceptions. Healthy adults also tend to have somewhat higher levels of anti-p40 antibodies than healthy children. These modest levels also tend to increase with age, perhaps related to the recently documented role of L1 retroelements in cellular senescence [23]. Our finding of anti-p40 autoantibodies in SLE has also been independently confirmed [30].

Second, we find that pSLE patients with active disease also have significantly elevated IgA autoantibodies in circulation against p40 compared to healthy controls, while patients with clinically inactive disease have intermediate levels. In contrast, adults with active SLE do not have IgA titers that differ from those of healthy controls (our unpublished observation). These data may indicate that IgA autoantibodies are uniquely elevated in the earliest stages of lupus disease and decrease to background levels with time. Since IgAs are particularly important for immunity on mucosal surfaces, their presence in pSLE patients may have implications for the anatomical location(s) where lupus pathogenesis is initiated.

Third, we find that p40 protein is present in a subset of circulating neutrophils both in pediatric and in adult SLE patients, particularly in those with active disease. The p40-positive neutrophils were not only present in the higher-density PMN fraction, but also in the lower-density PBMC fraction, suggesting that these p40-positive cells may include the unique subset of low-density granulocytes (LDGs) that have garnered considerable interest in SLE pathogenesis in recent years [31]. Of note, neutrophils are prevalent on mucosal surfaces and express more FcαR than other immune cells.

Fourth, we report that markers of neutrophil death by pathways that result in circulating DNA in complex with the neutrophil-specific enzymes MPO or NE are significantly elevated in pSLE patients with active disease compared to healthy controls. Samples from the same patients when their disease was inactive showed intermediate levels. Neutrophils from pSLE patients with active disease also had markedly elevated circulating S100A8/A9, indicative of neutrophil activation.

While presently only speculation, one could envision a scenario where the activation and death of p40-expressing neutrophils, some of them perhaps on mucosal surfaces, may be causally connected to increasing levels of IgG and IgA anti-p40 autoantibodies during active disease. The data in adult SLE patients are also compatible with this scenario. When complexed with released p40 (likely with bound RNA), the resulting IgG or IgA immune complexes may further activate neutrophils and induce their death by NETosis, escalating a detrimental feedback loop of potential importance in the pathogenesis of SLE. In this context, it might also be relevant that p40 exists in cells in a macromolecular assembly that includes Ro60, La-related proteins, snRNPs, and associated RNA [32–34]. Hence, p40 released from dying neutrophils may be associated with these proteins in addition to RNA.

The L1 encoded proteins ORF1p/p40 and ORF2p/p145 may play additional roles in SLE similar to the recently recognized role of L1 in cellular senescence and the associated production of IFNβ and age-related inflammation [23]. The mechanism for this involves the ORF2p protein, which is a reverse transcriptase that generates RNA:DNA hybrids and dsDNA that trigger the DNA sensor cGAS [10]. This same mechanism drives type I IFN production in certain forms of the interferonopathy known as Aicardi-Goutières syndrome [35], in which inhibitors of the reverse transcriptase reduced the interferon gene signature in a small clinical trial [36]. The production of IFNβ by senescent cells was also inhibited by reverse transcriptase inhibitors in tissue culture and in mice [23]. Whether these events also take place in p40-expressing neutrophils in SLE patients is currently under investigation in our laboratory. More specifically, we are measuring IFNβ and IFN-induced genes in neutrophils and are testing if reverse transcriptase inhibitors will block them. We are also using RNA sequencing to determine which L1 elements are expressed in SLE neutrophils.

## Conclusions

Our findings indicate that elevated L1 retrotransposon expression, primarily in neutrophils (among immune cells), is an early event in children developing pSLE, as well as in adults with a disease exacerbation. Active disease also associates with increased neutrophil activation and death, suggesting that immunogenic p40 may be released from these cells, resulting in the generation of IgG and IgA autoantibodies against p40. Limitations of our study include only two time-points of sampling, analysis of autoantibodies and neutrophils in blood only, but not bone marrow or tissue.

## Supplementary Information

**Additional file 1: Figure S1.** Flow cytometry and gating strategy for two representative pSLE patients. A, first patient: gating on live cells, then single cells, then expression of CD66b or CD19, then p40 in the CD66b+ and CD19+ populations. Note that p40 is predominantly found in the CD66b+ population with higher CD66b expression, presumably activated neutrophils. B, second patient: same gating strategy, showing CD66b+ and CD16+ populations.

## Data Availability

The datasets used and/or analyzed during the current study are available from the corresponding author on reasonable request.

## Abbreviations

ACPA: Anti-citrullinated protein antibodies
ANA: Antinuclear antibodies
BSA: Bovine serum albumin
CRP: C-reactive proteins
DMARD: Disease-modifying antirheumatic drugs
ELISA: Enzyme-linked immunosorbent assay
ESR: Erythrocyte sedimentation rate
HC: Healthy controls
HRP: Horseradish peroxidase
IFN: Interferon
JDM: Juvenile dermatomyositis
JIA: Juvenile idiopathic arthritis
LDGs: Low-density granulocytes
LINE-1: L1, long interspersed nuclear element
MHC: Major histocompatibility complex
MPO: Myeloperoxidase
NE: Neutrophil elastase
NSAID: Non-steroidal anti-inflammatory drugs
ORF1p: Open-reading frame 1-encoded protein
ORF2p: Open-reading frame 2-encoded protein
PBMC: Peripheral blood mononuclear cells
PBS: Phosphate-buffered saline
PMN: Polymorphonuclear cells
pSLE: Pediatric SLE
RF: Rheumatoid factor
SD: Standard deviation
SDS: Sodium dodecyl sulfate
SLE: Systemic lupus erythematosus
TMB: 3,3′,5,5′-tetramethylbenzidine
