# Supplementary figures and images for: IgG and IgA autoantibodies against L1 ORF1p expressed in granulocytes correlate with granulocyte consumption and disease activity in pediatric systemic lupus erythematosus
Source: Arthritis Res Ther. 2021 May 29;23:153. doi: 10.1186/s13075-021-02538-3 (PMC8164314; doi:10.1186/s13075-021-02538-3)

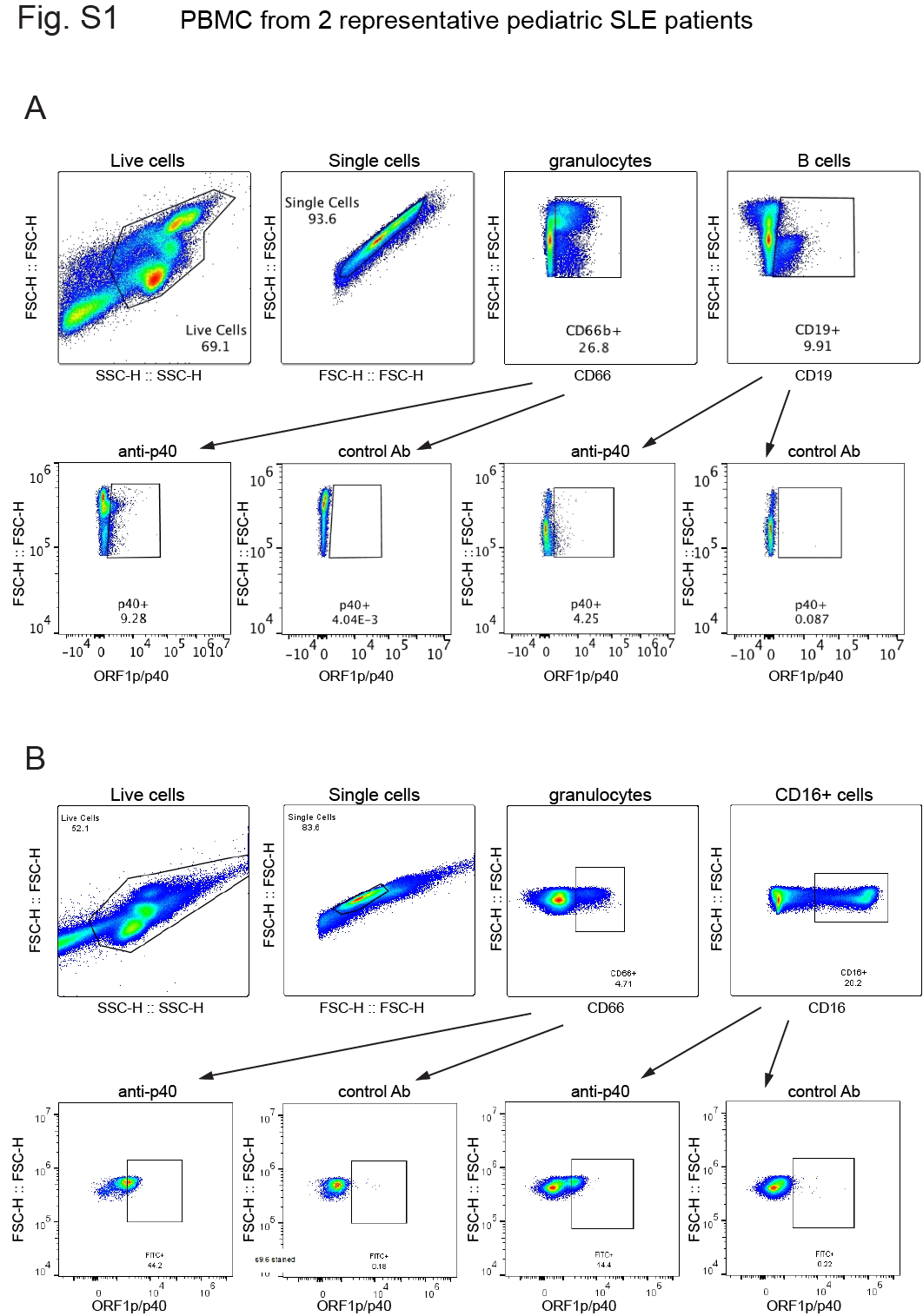

Supplement: Supplementary file 1 — Additional file 1: Figure S1. Flow cytometry and gating strategy for two representative pSLE patients. A, first patient: gating on live cells, then single cells, then expression of CD66b or CD19, then p40 in the CD66b+ and CD19+ populations. Note that p40 is predominantly found in the CD66b+ population with higher CD66b expression, presumably activated neutrophils. B, second patient: same gating strategy, showing CD66b+ and CD16+ populations. [file 13075_2021_2538_MOESM1_ESM.png]
